# Case Report: Interindividual variability and possible role of heterozygous variants in a family with deficiency of adenosine deaminase 2: are all heterozygous born equals?

**DOI:** 10.3389/fimmu.2023.1156689

**Published:** 2023-05-03

**Authors:** Federica Pulvirenti, Bianca Laura Cinicola, Simona Ferrari, Daniele Guadagnolo, Eleonora Sculco, Martina Capponi, Lorenzo Loffredo, Maddalena Sciannamea, Antonella Insalaco, Isabella Quinti, Fabrizio De Benedetti, Anna Maria Zicari

**Affiliations:** ^1^ Reference Centre for Primary Immune Deficiencies, Azienda Ospedaliera Universitaria Policlinico Umberto I, Rome, Italy; ^2^ Department of Maternal Infantile and Urological Sciences, Sapienza University of Rome, Rome, Italy; ^3^ Department of Molecular Medicine, Sapienza University of Rome, Rome, Italy; ^4^ Medical Genetics Unit, IRCCS Azienda Ospedaliero-Universitaria di Bologna, Bologna, Italy; ^5^ Department of Experimental Medicine, Sapienza University of Rome, Rome, Italy; ^6^ Department of Clinical, Internal Medicine, Anesthesiology and Cardiovascular Sciences, Sapienza University of Rome, Rome, Italy; ^7^ Division of Rheumatology, Bambino Gesù Children’s Hospital, IRCCS, Rome, Italy

**Keywords:** deficiency of adenosine deaminase 2, autoinflammatory disease, hypogammaglobulinemia, inborn errors of immunity, dada2, immunoglobulin

## Abstract

Deficiency of adenosine deaminase 2 (DADA2) is a rare systemic autoinflammatory disease, typically with autosomal recessive inheritance, usually caused by biallelic loss of function mutations in the *ADA2* gene. The phenotypic spectrum is broad, generally including fever, early-onset vasculitis, stroke, and hematologic dysfunction. Heterozygous carriers may show related signs and symptoms, usually milder and at an older age. Here we describe the case of two relatives, the proband and his mother, bearing an *ADA2* homozygous pathogenic variant, and a heterozygous son. The proband was a 17-year-old boy with intermittent fever, lymphadenopathies, and mild hypogammaglobulinemia. He also had sporadic episodes of aphthosis, livedo reticularis and abdominal pain. Hypogammaglobulinemia was documented when he was 10 years old, and symptoms appeared in his late adolescence. The mother demonstrated mild hypogammaglobulinemia, chronic pericarditis since she was 30 years old and two transient episodes of diplopia without lacunar lesions on MRI. *ADA2* (NM_001282225.2) sequencing identified both mother and son as homozygous for the c.1358A>G, p.(Tyr453Cys) variant. ADA2 activity in the proband and the mother was 80-fold lower than in the controls. Clinical features in both patients improved on anti-tumor necrosis factor therapy. An older son was found to be heterozygous for the same mutation post-mortem. He died at the age of 12 years due to a clinical picture of fever, lymphadenitis, skin rash and hypogammaglobulinemia evolving toward fatal multiorgan failure. Biopsies of skin, lymph nodes, and bone marrow excluded lymphomas and vasculitis. Despite being suspected of symptomatic carrier, the contribution of an additional variant in compound heterozygosity, or further genetic could not be ruled out, due to poor quality of DNA samples available. In conclusion, this familiar case demonstrated the wide range of phenotypic variability in DADA2. The search for *ADA2* mutations and the assessment of ADA2 activity should be considered also in patients with the association of hypogammaglobulinemia and inflammatory conditions, also with late presentation and in absence of vasculitis. Furthermore, the clinical picture of the deceased carrier suggests a possible contribution of heterozygous pathogenic variants to inflammation.

## Introduction

Deficiency of adenosine deaminase 2 (DADA2, MIM#615688) is a complex systemic autoinflammatory condition, typically with autosomal recessive inheritance, usually caused by biallelic loss of function mutations in the *ADA2* gene (adenosine deaminase 2, MIM*607575) also known as *CECR1* (Cat-Eye Syndrome Chromosome Region Candidate 1), mapping on the chromosomal region 22q11.1. The condition was first reported in 2014 by two independent groups, describing patients with vasculitis mimicking polyarteritis nodosa and multiple ischemic/hemorrhagic brain strokes (1, 2). In the following years, the clinical phenotype expanded, including a wider array of symptoms and variable severity (3). Vasculopathy with features of polyarteritis nodosa, leading often to early-onset stroke, is the most frequent and fearsome clinical manifestation (4). Moreover, mild to severe hematologic manifestations (5–8) and immunodeficiency have also been described and, in some cases, can be the only manifestation (9).

Incomplete penetrance has been described in asymptomatic individuals bearing biallelic pathogenic variants (3, 10–13). The onset of the disease typically occurs in the first decade of life, but adult-onset forms have also been reported without differences in clinical and immunological phenotype (14, 15). Heterozygous carriers may show related signs and symptoms, usually milder and at an older age (16). Currently, TNF inhibitors are the treatment of choice to reduce the risk of vascular events and control inflammation but seem not effective for immunodeficiency and the hematological manifestations, which require hematopoietic stem cell transplantation (16–18).

ADA2, together with ADA1, plays a role in purine metabolism and adenosine homeostasis, catalyzing the hydrolytic deamination of adenosine and 2-deoxyadenosine to inosine and deoxyinosine (19, 20). The deaminase function of ADA2 under physiological conditions is limited due to an elevated Km (low affinity) for the substrate. This explains the predominant role of ADA1 as the regulator of adenosine concentrations in physiological circumstances. Reduced or absent levels of ADA2 cause impairment of adenosine catabolism at the site of inflammation, which leads to chronically increased adenosine levels and, consequently, chronic inflammation (21). In addition to reducing extracellular adenosine levels, ADA2 may act in the extracellular space as a growth factor contributing to maintaining endothelial integrity (22, 23). ADA2 is primarily expressed by monocytes and other cells of the myeloid lineage, its activity is mainly detected in blood (24), and it is required for a proper monocyte-T cells interaction (25). However, the role of ADA2 in immune processes, especially in the adaptive compartment and in the development of hematologic defects, still needs to be clarified.

Biallelic pathogenic variants in *ADA2* and deficient levels of ADA2 enzymatic activity in the peripheral blood are diagnostic of DADA2 (16). The functional assessment of ADA2 activity can be important, as some pathogenic variants, including variants in non-coding regions or intragenic deletions and duplications, are not conventionally identified by targeted approaches, namely Sanger Sequencing and Next Generation Sequencing (NGS) panels or exome sequencing (26).

In addition, *ADA2* displays a high rate of Variants of Uncertain Significance (VUS) (3, 10). These difficulties in achieving a molecular diagnosis, the reduced penetrance, and variable expressivity may affect clinical management (3, 15, 18). Environmental and epigenetic modifiers could explain this intrafamilial variability, but more studies are needed to elucidate this aspect (10). Here we report the description of a family carrying the same variant but presenting a different clinical expression of the disease and a different outcome.

## Case reports

A 17 years-old male (III:2) was referred to the Pediatric Immunological Division of Policlinico Umberto I in Rome presenting with recurrent fever. The patient was born at term by spontaneous delivery after physiological pregnancy. The neonatal period was normal. He never had severe infections. He suffered from sporadic episodes of recurrent aphthosis and livedo reticularis after exposure to cold. At the age of 16, he had a sudden onset of serotinous fever, mild arthralgias of the lower limbs, sporadic episodes of abdominal pain with diarrhea, and an episode of maculopapular rash on his hands. Fever raised to the maximum temperature of 38.5°C and was initially sporadic. Three months before our first evaluation, fever episodes had developed a recurrent pattern of 15-20 days, with a duration of about ten days. When he was referred to our hospital, he was afebrile with stable vital signs. Livedo reticularis was noted on his limbs. Laboratory tests showed normal white blood cells, elevated acute-phase reactants (APRs), and elevated fibrinogen and D-dimers (5.46 g/L and 565 microg/L). Decreased immunoglobulin (Ig) M and IgA levels were observed, with normal IgG, as well as defective immunization response to HBV and Pneumococcal Polysaccharides, with normal levels of total B cells and reduced memory B cells. Serum autoantibodies were negative (**Table 1**). Microbiological tests were negative, including viral serologies, bacterial cultures, QuantiFERON, blood culture, urine culture, and parasitological examination of feces. Fecal calprotectin was also negative. A CT scan revealed bilateral lateral-cervical and submandibular lymphadenopathies with reactive features without hepatosplenomegaly. The patient was the second child of apparently non-consanguineous parents who, however, originated from the same small Italian town with less than seven hundred inhabitants and shared the same surname.

**Table 1:** Laboratory results of the patient and his relatives.

	Father II:5 c.1358 A>G; p.(Tyr453Cys) (het)	Mother II:6 c.1358 A>G; p.(Tyr453Cys) (hom)	Proband III:2 c.1358 A>G; p.(Tyr453Cys) (hom)	Brother III:1 c.1358 A>G; p.(Tyr453Cys) (het)
**GR (4.3-5.9)/Hb (13.5-16.5)/PLT (150-450) x10^3^ cell/mm^3^**	4.95/15.5/251	4.60/11.9*/174	5.48/13.6/291	4.64/11.5/250
**WBC (4.4-11.3)/N (1.8-7.7)/L (1.0-4.8) x10^3^ cell/mm^3^**	6.48/3.98/1.83	5.13/3.50/1.01	7.15/4.30/1.97	14.46/9.99/1.50
**ESR sec (<20)**	2	9	25	38
**RCP mg/dl (<0.5)**	1.0	1.2	5.99	6.11
**Ferritin ng/mL (30-400)**	234	280	200	9456
**Gamma globulins % (11-18)**	13.6	11	12	10.7
**IgG mg/dL (700-1600)**	1000	630	851	487
**IgG1 mg/dL (37-1280)**	680	488	603	NA
**IgG2 mg/dL (106-610)**	342	123	158	NA
**IgG3 mg/dL (18-163)**	69.3	21	34	NA
**IgG4 mg/dL (4-230)**	79.1	65	56	NA
**IgA mg/dL (70-400)**	304	32	52	79.6
**IgM mg/dL (40-230)**	47	21	20	68
**IgE kUI/l (<100)**	0	11	12	60.2
**CH50% (70-140)**	97	98	96	95
**ANA/ENA/anti-dsDNA**	neg/neg/neg	neg/neg/neg	neg/neg/neg	neg/neg/neg
**C3 g/l (80-185)/C4 g/l (15-53)**	1.2/0.2	1.3/0.2	1.6/0.3	1.5/0.4
**Rheumatoid factor UI/mL (<15)**	neg	neg	neg	neg
**CD3+ % (54-85)/CD4+ % (31-59)/CD8%+ (10-38)/CD19+ % (5-22)/CD16 + 56+ % (4-28%)**	70/53.2/14.3/12.6/17.6	62.6/38.1/17.6/17.3/19.2	66.4/41.5/18.2/15.4/16.2	76.6/42.7/31.3/10/11.4
**B cells subset, % of CD19+ Transitional/Mature/Memory/Plasma cells**	6,8/57,1/28,8/0,62	9.4/82.2/7.7/0.3	6/86.3/7.3/0.4	NA
**ADA2 activity, mU/g (biallelic LOS carriers 4.7 ± 4.8; het LOS carriers 55.3 ± 22; controls 130 ± 53.2)**	60.1	1.6	2.1	NA

RBC, red blood cells; Hb, hemoglobin; PT, platelets; WBC, white blood cells; N, neutrophils; L, lymphocytes; ESR, erythrocyte sedimentation rate; CRP, C reactive protein; Ig, immunoglobulin; HBsAb, Hepatitis B s antibodies; CH50, Complement activity assay; ANA, antinuclear antibody; ENA, extractable nuclear antigen; ds DNA, double stranded DNA; C, complement; RF, rheumatoid factor; LOS, loss of function; neg, negative. Referral values are shown in the bracket.

The patient’s father (II:5) was 47 years old and reported to be healthy. The mother’s brother and the father’s four siblings had a negative past medical history for vascular or autoimmune diseases and immunodeficiency. The patient’s mother (II:6) was 51 years old and had a past medical history positive for hypogammaglobulinemia (IgA and IgG1 and IgG2 deficiency), chronic pericarditis discovered in the first postpartum, autoimmune thyroiditis, and two previous episodes of transient diplopia with normal CNS MRI findings.

**Figure 1:**
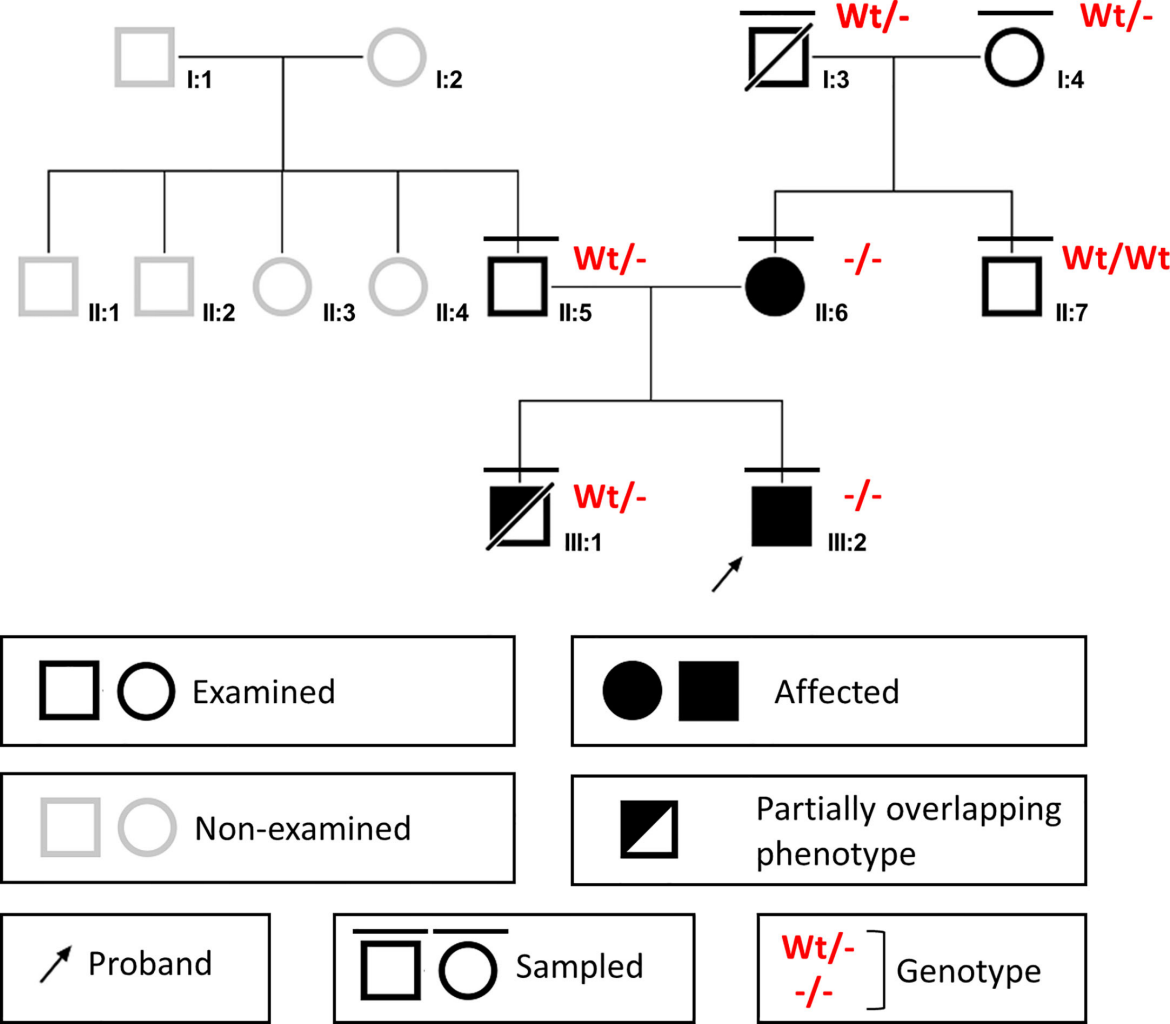
Pedigree of the family.

The patient had an older brother (III:1) who previously died from multiorgan failure at 12 years of age (**Figure 1**). The boy was previously healthy, being admitted to the hospital with a fever, skin rash and mild pleuro-pericarditis associated with low IgG levels and elevated APRs. The clinical picture evolved rapidly towards a picture of DRESS (Drug Rash with Eosinophilia and Systemic Symptoms)-like syndrome, respiratory failure, and seizures. Microbiological tests were negative, including blood cultures, qRT PCR for EBV, CMV, Mycobacteria, *Leishmania* spp., and serology for herpes viruses, enterovirus, *Salmonella* and *Brucella* spp., *Borrelia* spp., *Echinococcus* spp., *Mycoplasma* spp., and HIV. A CT scan revealed a pulmonary opacity with pleural effusion, hepatosplenomegaly, and abdominal lymphadenopathy with reactive features. Laboratory tests revealed hyper eosinophilia (>8000 cell/mm3) and high serum levels of ferritin (>10.200 ng/mL), LDH (2900 UI/L), triglycerides (920 mg/dL). Skin, bone marrow and lymph-nodes biopsies excluded lymphomas and vasculitis. Despite treatment with systemic steroids and broad-spectrum antibiotics and antifungal drugs the boy died. The autopsy revealed a picture of eosinophilic myopericarditis and interstitial pneumonia, with secondary erythrophagocytosis in the spleen, liver, lymph nodes and bone marrow, acute tubular necrosis, and brain edema.

Given the family history of hypogammaglobulinemia and a family history positive for early death, inherited inborn errors of the immune system (IEIs) were considered. The NGS analysis for a panel of 49 genes involved in monogenic disease with defects in humoral immunity (**Supplementary Materials**) was proposed for the proband, and written informed consent was gathered. The NGS analysis was performed on the Ion Torrent S5 system (Thermo Fisher Scientific). The coverage was 88.42% at 500x read depth and 98.98% at 20x depth. Sequence data analysis was performed with the Ion Reporter v5.12 software (Thermo Fisher Scientific). Variants with a minor allele frequency >0.01 were filtered out. Only nonsynonymous variants and splice site variants were retained. A visual analysis of the reads was performed, including candidate variants mapped against the reference genome (version GRCh37/hg19). Variant validation was performed by Sanger sequencing on the 3730 Genetic Analyzer platform (Thermo Fisher Scientific). The NGS analysis identified the c.1358A>G, p.(Tyr453Cys) variant in exon 9 of the *ADA2* gene (NM_001282225.2) in homozygosity. The variant was reported once in ClinVar as pathogenic and has been described before in the literature. It can be classified as Likely Pathogenic according to the American College of Medical Genetics and Genomics guidelines, applying the PM5 moderate, PP2 moderate, and PM3 moderate criteria (27). The variant was found in homozygosity also in the proband’s mother. Genetic analysis was also performed on DNA extracted by Formalin-Fixed Paraffin-Embedded (FFPE) tissues of the deceased brother of the proband (III:1), who was found to be heterozygous for the variant. The patient’s father was also confirmed to be heterozygous. The proband’s maternal grandparents, I:3 and I:4, were also identified as heterozygous carriers. They both did not display signs or symptoms of autoinflammation, immune dysregulation or immune deficiency. I:3 later died due to unrelated conditions.

Serum ADA2 level was assessed on the proband (III:2) by dried blood spot and found to be 2.1 mU/g (reference values: *ADA2* biallelic loss-of-function variant carriers 4.7 ± 4.8; *ADA2* heterozygous carriers 55.3 ± 22 mU/g; controls 130 ± 53.2 mU/g). A diagnosis of DADA2 was made, and the patient was treated with subcutaneous etanercept 25 mg weekly. Brain magnetic resonance imaging did not identify chronic ischemic lesions or other vascular abnormalities. At the last follow-up after etanercept therapy for 12 months he remained asymptomatic without fever, abdominal symptoms, and vascular events. In addition, his APR level had normalized.

Serum ADA2 level from the proband’s mother (II:6) was measured by dried blood spot and found to be 1.6 mU/g, and she was also diagnosed as having DADA2. Laboratory tests showed normal WBC and mild hypogammaglobulinemia (IgG 630 mg/dL, IgA 32 mg/dL, and IgM 21 mg/dL), with reduced memory B cells and no serum autoantibodies (**Table 1**). A CT scan revealed thymic residual, bilateral axillary and cervical lymphadenopathies, hepatomegaly, and mild pericardial effusion. She initially refused to perform a Brain MRI and to be treated with a TNF inhibitor. One year later, she developed remittent fever with worsening pericardial effusion. A CT scan also revealed the presence of pleural effusion without lung abnormalities. Laboratory tests showed elevated APRs. Microbiological tests were negative, including qRT PCR for viruses, bacterial cultures, QuantiFERON, blood and urine culture. The patient was initially treated with colchicine and intravenous methylprednisolone followed by oral prednisolone, with rapid improvement of symptoms and reduced APRs. She then switched to adalimumab 40 mg every two weeks in the following weeks, and her condition has improved. Soon after, she was infected by SARS-CoV-2. She presented fever and cough and was treated by sotrovimab. She developed a relapse of fever with pleuro-pericarditis and high APRs two-weeks thereafter, treated by intravenous steroids. Moreover, one year after the DADA2 diagnosis she started immunoglobulin replacement treatment due to the progressive reduction of IgG serum levels. Serum *ADA2* levels were also assessed in the heterozygous father of proband, and the result was 60.1 mUI/g, comparable to *ADA2* carriers.

The *ADA2* familial variant segregation analysis by Sanger sequencing was extended to the proband’s maternal grandparents (I:3 and I:4) and to the maternal uncle (II:7), after genetic counseling with written informed consent. Their past medical histories were negative for neurological, hematological, or immunological relevant issues. The segregation analysis identified the heterozygous c.1358A>G variant in both the maternal grandparents, whereas the maternal uncle was found homozygous for the wild-type allele (**Figure 1**).

**Figure 2:**
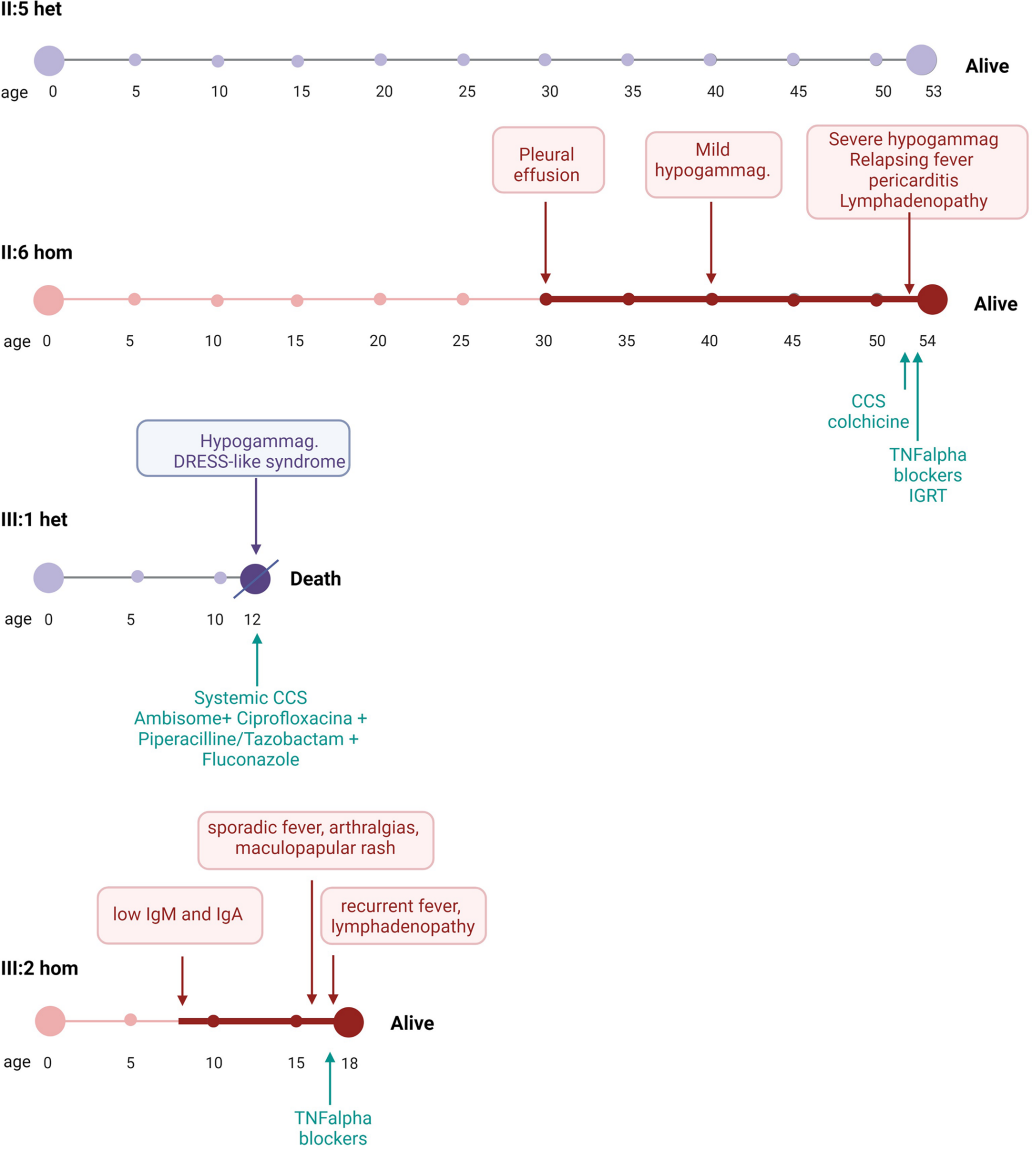
Clinical expression, treatment, and outcome in family members carrying biallelic (red timelines) or monoallelic (purple timelines) pathogenic variants of *ADA2*. For each person, the period following the first presentation of disease is represented by a bold bar. het, heterozygous; hom, homozygous; CCS, corticosteroids; TNF, tumor necrosis factor; IGRT, immunoglobulin replacement therapy.

Age of onset, clinical expression, treatment, and outcome in the family members carrying biallelic or monoallelic pathogenic variants of *ADA2* is summarized in **Figure 2**.

## Discussion

DADA2 is a rare autoinflammatory disease caused by homozygous or compound heterozygous loss-of-function pathogenic variants in the *ADA2* gene. The disease affects multiple organs with a highly heterogeneous clinical picture, usually presenting before the age of 10 years (1, 2, 4, 16). However, non-specific clinical features or late-onset presentation may delay its diagnosis until adulthood (16). Familial screening is essential to diagnose DADA2 before the onset of symptoms, possibly before the occurrence of strokes and to start early treatment with TNF inhibitors.

For the genetic diagnosis, the identification of biallelic pathogenic variants in *ADA2* is required. Testing the ADA2 protein level is useful in case of a clinical suspicion to guide the interpretation of genetic results (11, 12). The most commonly identified disease-causing variants of the *ADA2* gene are p.Gly47Arg (p.G47R), p.Arg169Gln (p.R169Q), p.Gly47Ala (p.G47A), and p.Tyr453Cys (p.Y453C), being described in DADA2 patients both in homozygous or compound heterozygous state (1, 2).

Apart from the amount of residual ADA2 activity, identifying some disease patterns has led to the idea that a possible genotype/phenotype correlation exists but has not been established so far (28, 29). Familiar cases of DADA2 provide a unique insight into its disease’s wide range of phenotypic variability. Here we described the clinical picture of members of an Italian family carrying the same mutation but presenting a different clinical expression of the disease and different outcome.

Mild hypogammaglobulinemia was the common denominator of this family, shared by both the affected individuals and the 12-years old boy bearing the monoallelic pathogenic variant, who died in his adolescence. The impairment of the B-cells compartment in DADA2 was clear since the initial descriptions (1). Since then, immunodeficiency has been revealed as a significant but inconstant aspect of the DADA2 clinical picture, with hypogammaglobulinemia being reported in about 60% of the patients, with 2/3 of them having recurrent respiratory infections (9). Vice versa, screening of cohorts with antibody deficiency using next generation sequencing and targeted Sanger sequencing revealed a prevalence of biallelic pathogenic variants in *ADA2* amounting to 6%, all patients identified having associated autoimmunity or dysregulation (9). A defect of B cells terminal maturation has been confirmed by several authors and may reflect a role of *ADA2* in the bone marrow microenvironment (30). In particular, the B cell appears to be less able to undergo Ig class-switching recombination and more prone to autoreactivity and apoptosis than in immunocompetent subjects (4, 31). Moreover, DADA2 naïve B-cells also display a reduced capacity to respond to stimulation by TLR 9 agonist, CD40L, and anti-human immunoglobulins, suggesting a proliferation defect induced by the loss of *ADA2* (31). About the T-cell pool, CD4+ and CD8+ T cells can be diminished, with T follicular helper cells showing a functional impairment in IL-21 production, possibly contributing to an impaired B cell class-switch recombination (31).

Beside the immunoglobulin defect, both the 17-year-old proband and his 51-year-old mother presented late-onset nonspecific findings making the diagnosis challenging. Despite harboring the same homozygous pathogenic variant, they showed distinct clinical phenotypes, differing clinical manifestations and onset age, sharing only the antibody defect. In detail, whereas the proband displayed recurrent fever and other mild symptoms of inflammation in his late adolescence, his mother was otherwise well until age 30, when she was diagnosed with chronic pericarditis, with fever and worsening serositis and hypogammaglobulinemia only in her fifties. Phenotypic variability in age of the onset and severity of symptoms has been reported in individuals with the same mutation, even in the same family (32), suggesting the presence of additional disease modifiers, such as susceptibility alleles in other *loci* and epigenetic or environmental factors (3, 16). To further complicate the picture, the older brother of the proband also developed hypogammaglobulinemia and signs consistent with systemic inflammation due to DADA2, despite carrying only a monoallelic pathogenic variant.

It has already been reported that, although most patients have biallelic loss of function mutations in the *ADA2* gene, in some cases sequencing may result in the identification of a pathogenic variant on a single allele (3). Heterozygous patients with a mild phenotype have been described with an intermediate profile of ADA2 activity (3), while subjects with a full clinical picture displayed a complete absence of enzymatic activity, as occurred in patients with biallelic mutations (11). In this subgroup, the presence of a second *ADA2* pathogenic variant not detected by conventional sequencing and genetic testing should be considered (3). Moreover, heterozygous asymptomatic family members have been shown to display enzymatic activity in the carrier range (11, 33).

ADA2 enzyme activity measurement may then offer a useful tool for screening for DADA2 both in patients with a suspicious clinical history and in unaffected siblings before to genetic testing (34). If the *ADA2* plasma activity is in the normal range, an alternative molecular diagnosis should be considered (27). The question of how to proceed with low or absent ADA2 activity in seemingly unaffected siblings remains unresolved, since the level of enzyme activity does not always correlate with the clinical severity (34, 35). Importantly, whether heterozygous pathogenic variants predispose to DADA2 manifestations remains to be investigated, since some carriers of pathogenic *ADA2* mutations with ADA2 activity levels in the carrier range show mild and/or late-onset manifestations (12, 16). To explain these findings, some authors have proposed a possible dosage-sensitivity mechanism for *ADA2*, putatively unmasked by other genetic modifiers or environmental triggers (16). These studies also showed that the function and phenotype of peripheral lymphocytes from heterozygous symptomatic carriers were intermediate to that of healthy donors and *ADA2*-deficient patients (30). Unfortunately, enzymatic test has not been performed in the symptomatic heterozygous carrier in this family (III:1) since he died before the DADA2 first report, in 2014. Therefore, a conclusive diagnosis for this subject cannot be made. However, the enzymatic test has been performed on the father, who was an asymptomatic heterozygous carrier, revealing reduced ADA2 activity. In the deceased boy the contribution of a further *ADA2* variant in compound heterozygosity, or further genetic and environmental factors, could not be ruled out. Importantly, we cannot exclude the possibility of an additional autosomal recessive disorder, due to his parents’ possible consanguinity and peculiar geographical origin from a genetic isolate.

Standard molecular genetic testing for DADA2 is commonly conducted using conventional Sanger sequencing for targeted genes and by NGS–based targeted panels or exome sequencing (26, 34, 35). However, these routine techniques may miss structural and non-coding causal variants, small deletions, and duplications. Patients with suspected clinical phenotypes and low ADA2 activity but without identifiable mutations or those bearing monoallelic pathogenic variants, *ADA2* variants might be investigated by other techniques such as Chromosomal Microarrays, Multiplex Ligation-dependent Probe Amplification, quantitative Polymerase Chain Reaction, or whole genome sequencing (3, 35). It is reported that *ADA2* sequence analysis can identify up to 97% of known pathogenic *ADA2* alleles, while only 3% of causative variants can only be identified by targeted deletion/duplication analysis (36). Unfortunately, such investigation could not be performed on DNA extracted from FFPE tissues of III:1, the deceased brother of the proband, due to the low quantity and suboptimal quality of the extracted DNA, in absence of other available samples.

In conclusion, we described a family in which the DADA2 phenotype manifested prominently as antibody deficiency and immune dysregulation rather than vasculopathy and early-onset stroke. Integrating clinical pictures, quantitative ADA2 assays and molecular data is pivotal in achieving a diagnosis and clinical management of individuals and families. Furthermore, the severe phenotype of the deceased carrier poses questions on the possible clinical relevance of the heterozygous variant and its contribution to inflammation, likely in addition to other genetic and environmental factors. Given the limitations imposed in this case by the scarcity of the available material, further research is needed to confirm or exclude this possible association.

## Ethics statement

Written informed consent was obtained from the participant/patient(s) for the publication of this case report.

## Author contributions

FP and BLC wrote the first draft. SF performed the genetic analysis. SF and DG interpreted the genetic data. FP, BC, IQ, AMZ, AI, and FDB treated and take care of the follow up of patients. SF, DG, ES, MC, LL, MS, AI, IQ, FDB, and AMZ provided critical comments and editorial suggestions for revisions. All authors contributed to the article and approved the submitted version.
