# Supplementary material for: Case Report: Interindividual variability and possible role of heterozygous variants in a family with deficiency of adenosine deaminase 2: are all heterozygous born equals?
Source: Front Immunol. 2023 May 3;14:1156689. doi: 10.3389/fimmu.2023.1156689 (PMC10188974; doi:10.3389/fimmu.2023.1156689)
Supplement: Supplementary file 1 [file Table_1.docx]

**Supplementary materials**

List of 49 genes included in the IEIs panel

*ADA, AICDA, ATM, BLNK, BTK, CASP8, CASP10, CD19, CD3G, CD40, CD40LG, CD79A, CD79B, CD81, CECR1, CR2, CTLA4, DKC1, FAS, FASLG, ICOS, IKZF1, IL2RA, KRAS, LIG1, LRBA, MAGT1, MS4A1, NFKB1, NFKB2, NFKBIA, NRAS, OAS1, PIK2CD, PIK3R1, PLCG2, PRF1, PRKCD, RAG1, RAG2, SH2D1A, STAT1, STAT3, TCF3, TNFRSF13B, TTC7A, UNG, WAS, XIAP*.
